# Low β-carotene bioaccessibility and bioavailability from high fat, dairy-based meal

**DOI:** 10.1007/s00394-024-03423-w

**Published:** 2024-05-16

**Authors:** Johanita Kruger, Nadine Sus, Andrea Moser, Sophie Scholz, Guenther Adler, Sascha Venturelli, Jan Frank

**Affiliations:** 1 Institute of Nutritional Sciences, University of Hohenheim, Garbenstraße 28, 70599 Stuttgart, Germany; 2 Department of Consumer and Food Sciences and Institute of Food Nutrition and Well-Being, University of Pretoria, Hatfield, Private Bag X20, Pretoria, 0028 South Africa

**Keywords:** Bioaccessibility, Cellular uptake, Divalent minerals, Food matrix, Lipid profile, Micellization, Dairy, BCMO1

## Abstract

**Purpose:**

The original aim of the study was to determine, in a double-blind 3-arm crossover human trial (n = 7), the effect of supplemental levels of iron (25 mg) and zinc (30 mg) on β-carotene (synthetic) bioavailability (10 h postprandial). However, despite the high dose of supplemental β-carotene (15 mg) consumed with the high fat (18 g), dairy-based breakfast test meal, there was a negligible postprandial response in plasma and triglyceride rich fraction β-carotene concentrations. We then systematically investigated the possible reasons for this low bioavailability of β-carotene.

**Methods:**

We determined (1) if the supplemental β-carotene could be micellised and absorbed by epithelial cells, using a Caco-2 cell model, (2) if the fat from the test meal was sufficiently bioavailable to facilitate β-carotene bioavailability, (3) the extent to which the β-carotene could have been metabolised and converted to retinoic acid/retinol and (4) the effect of the test meal matrix on the β-carotene bioaccessibility (in vitro digestion) and Caco-2 cellular uptake.

**Results:**

We found that (1) The supplemental β-carotene could be micellised and absorbed by epithelial cells, (2) the postprandial plasma triacylglycerol response was substantial (approximately 75–100 mg dL^−1^ over 10 h), indicating sufficient lipid bioavailability to ensure β-carotene absorption, (3) the high fat content of the meal (approximately 18 g) could have resulted in increased β-carotene metabolism, (4) β-carotene bioaccessibility from the dairy-based test meal was sixfold lower (p < 0.05) than when digested with olive oil.

**Conclusion:**

The low β-carotene bioavailability is probably due to a combination of the metabolism of β-carotene to retinol by BCMO1 and interactions of β-carotene with the food matrix, decreasing the bioaccessibility.

**Trail registration:**

The human trail was retrospectively registered (ClinicalTrail.gov ID: NCT05840848).

**Supplementary Information:**

The online version contains supplementary material available at 10.1007/s00394-024-03423-w.

## Introduction

Optimal carotenoid bioavailability is important as carotenoids function as provitamin A and/or antioxidants. β-carotene, from plant-based diets is often the main source of vitamin A, either due to socio-economic status (cannot afford animal products) or personal choice (vegetarianism/veganism). Epidemiological studies have shown a positive link between dietary intake as well as tissue concentrations of carotenoids and a lower risk of chronic disease [1], and dietary or circulating beta-carotene has also been inversely associated with risk of all-cause mortality [2].

The initial aim of the study was to determine the effects of dietary supplements of iron (25 mg) and zinc (30 mg) on β-carotene and lipid bioavailability in healthy male volunteers. Decreased bioaccessibility and cellular uptake observed in In vitro studies [3–5], suggest that supplemental levels of divalent minerals could inhibit the bioavailability of β-carotene in humans [3–5]. The decreased bioaccessibility and cellular uptake in these studies were mainly attributed to the divalent minerals capacity to precipitate bile acids and decrease the stability of physiological mixed micelles. Three in vivo studies that have investigated the effects of supplemental levels of calcium and iron on the bioavailability of carotenoids in healthy adults had contradictory findings [6–8].

However, despite the high dose of β-carotene consumed with the test meal (15 mg), there was a negligible postprandial response in plasma and triglyceride rich fraction (TRF) β-carotene concentrations. We then systematically investigated the possible reasons for the low bioavailability of β-carotene observed. For β-carotene to be bioavailable various steps are required, including (1) release from the food matrix, (2) incorporation into physiological mixed micelles, (3) uptake by intestinal epithelial cells, and (4) packaging into chylomicrons that are secreted into the lymphatic system [9].

We evaluated the effects various factors could have on these steps, resulting in low bioavailability. First, we determined if the β-carotene was released from the capsule and if the supplemental β-carotene was in a form that could be included in micelles and be absorbed by epithelial cells. The crystalline form of carotene has been found to decrease both the bioaccessibility and bioavailability of carotenes [10]. Secondly, by evaluating the post-prandial triacylglycerol (TAG) response, we evaluated if the fat from the test meal was sufficiently digested and absorbed to facilitate β-carotene absorption and/or transport into the lymphatic system. The amount of fat (5–28 g) in a meal and its digestion is critical to ensure optimal micellization and absorption of β-carotene [9, 11, 12]. We then investigated the extent to which the β-carotene could have been metabolised and converted to retinoic acid/retinol during absorption, by evaluating the post-prandial retinol response. Dietary factors can affect the activity of BCMO1, resulting in more or less of the absorbed β-carotene being converted [13]. Lastly, using in vitro digestion and Caco-2 cell culture models, we evaluated the effect of the test meal matrix on the β-carotene bioaccessibility and cellular uptake.

## Materials and methods

### Sample size calculation and study population

The required power for the study was calculated based on the area under the plasma β-carotene concentration–time curve (AUC). The study power was calculated based on the following formula: n > F (σ/d)^2^, where n = number of subjects, σ = standard deviation of the dependent primary outcome measure, d = difference between control and treatment classified as significantly different in the literature. The corresponding F-value of 20.86 at P < 0.025 and 99% power were used [14].

In a comparable study by (Goltz et al. 2013), an AUC of 22.6 ± 7.6 nmol L^−1^ h could be determined after intake of 20–30 mg β-carotene from one meal (n = 6). Based on these reference values, a mean value of 22.6 nmol L^−1^ h and a standard deviation of 7.6 nmol L^−1^ h was used in the calculation. A 50% reduction in bioavailability would mean a biologically relevant effect, which is why a significant difference of 11.3 nmol L^−1^ h was used for the calculation. Thus, n > 20.86 × (7.6/11.3)^2^ resulted in a number of at least 9.44, rounded up to ten subjects. Since this was a cross-over study with no gender effects and considering average drop-out rates observed in previous studies, 12 participants were included in the study.

After screening 23 males, a total of twelve healthy male participants (aged 18–45 years old) with routine blood chemistry values within the normal ranges (Supplemental Table A1) and who met the inclusion criteria were included in this study. Women were excluded from the study because the concentrations and distribution of carotenoids in the lipoprotein fractions fluctuate throughout the menstrual cycle [15, 16]. Other exclusion criteria were overweight (BMI > 25 kg m^−2^), metabolic and endocrine diseases, drug abuse, use of dietary supplements or any form of medication, smoking, frequent alcohol consumption (> 20 g ethanol day^−1^), adherence to a restrictive dietary regimen, physical activity of more than 5 h/week, participation in a clinical trial within the past 3 months prior to recruitment, or a known intolerance against β-carotene, iron and/or zinc supplements. All participants were asked to maintain their regular lifestyles and usual extent of physical activities during the study period. The study protocol (AZ: F-2017-039) was approved by the ethics committee of the State Medical Society of Baden-Wuerttemberg, Germany, and was in conformance with the Declaration of Helsinki. Written informed consent was obtained from all participants before inclusion in the trial.

**Table 1:** Composition and time of consumption (relative to the intake of β-carotene preparations at 0 h) of standardised meals

Meal	Time (h)	Composition
Dinner	−10	Refined white bread, camembert, butter, macadamia nut, coconut yoghurt
Breakfast	0	Refined white bread roll, medium fat cream cheese, Greek yoghurt with honey
Lunch	4	Pasta with white cheese sauce, bread roll, yoghurt ice cream
Snack	6	Coffee or tea, coconut macaroons
Dinner	8	Refined white bread, butter, potato salad, almonds, white hazelnut yoghurt

### Study design and dosage information

The study followed a double-blind crossover design with three study arms separated by one-week washout periods. In short, the participants were asked to follow a diet low in carotenoids by avoiding all orange, yellow, red and green fruits and vegetables for four days. This was followed by three days of a strictly carotenoid-free diet, which only allowed foods from a specified list (Table 1). On each study day, β-carotene was administered in the morning after a > 10 h overnight fast (Supplemental Fig. A1). All participants orally ingested, in random order, a single dose of 15 mg synthetic β-carotene in a soybean/corn oil matrix (BIOVEA) with either a placebo (empty capsule), 25 mg iron (FeSO_4_; Woerwag Pharma GmbH & Co. KG, Boeblingen, Germany) or 30 mg zinc (ZnSO_4_; Woerwag Pharma) capsule. The iron and zinc concentrations were chosen considering the tolerablr upper limit of intake, suggested maximum levels in supplements as well as average iron and zinc contents of supplements in the Europe market. A standardised dinner was provided on the evening before the trial and standardised meals were provided during the entire intervention day (Table 1). On the first of three study days, if the amount of food (weight or volume) consumed by each participant varied at all from the standardised meal, it was recorded and the same amounts provided during the following two arms to ensure similar food, and especially fat consumption. Water was available unrestricted for consumption throughout the day. Blood samples were drawn from an indwelling venous cannula and collected at 0 h directly before β-carotene supplementation and then every hour for 10 h.

**Fig. 1:**
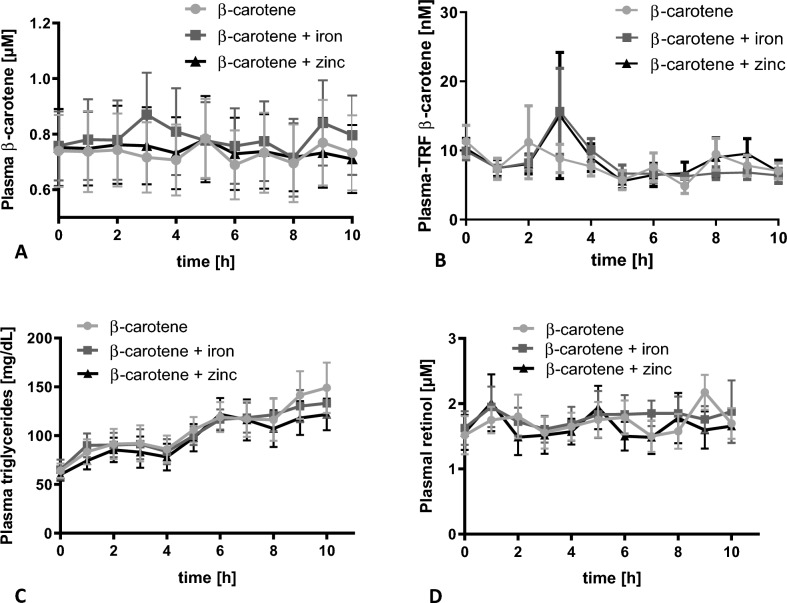
Mean (**A**) plasma β-carotene, (**B**) TRF β-carotene, plasma (**C**) TAG and (**D**) retinol concentrations (mean ± SD) over the course of 10 h after a single oral dose of 15 mg β-carotene together with a placebo, iron sulphate (25 mg iron) or zinc sulphate (30 mg zinc). Differences between groups (n = 6) were analysed by one way ANOVA, but no significant differences were observed

### Blood sampling and processing

For the determination of plasma concentrations of β-carotene, LDL- and HDL-cholesterol, and triacylglycerols (TAG), blood was collected in tubes containing EDTA (Sarstedt AG & Co, Nuebrecht, Germany) and immediately centrifuged (3000×*g*, 10 min, 4 °C). From the obtained plasma samples, three aliquots were stored at − 80 °C until further analysis and the rest ultracentrifuged to obtain the triacylglycerol-rich fraction (TRF). For the analyses of liver and kidney function markers, plasma and serum were obtained from blood sampled at the 0- and 4-h time points.

The TRF was prepared according to [11]. Briefly, plasma (3.5 mL) was transferred to an ultracentrifuge tube and carefully overlaid with 8 mL 1.3% sodium chloride and then ultracentrifuged (Beckman Coulter, OptimaTM L-80 XP Ultracentrifuge) using a swinging bucket rotor (SW41Ti) at 150,000×*g* for 1 h at 4 °C. Afterwards, the TRF was isolated by transferring the upper ~ 6 mL by pipette, which was then overlaid with nitrogen gas to minimize oxidation and stored at −80 °C until extraction.

### β-Carotene analysis in plasma and TRF

The plasma samples were randomly extracted and analysed by HPLC according to [17]. Briefly, 40 µL plasma was extracted with an ethanol/*n*-butanol mixture (50:50) containing apo-80-carotenal-methyloxime [12 µL/100 mL; Fluka Analytical (Merck Group KGaA), Darmstadt, Germany] as internal standard. After centrifugation, the clear supernatant was analysed by HPLC.

The TRF was extracted and analysed by HPLC [17]. For the extraction, 100 µL apo-80-carotenal-methyloxime (12 µL/100 mL) and 2 mL ethanol (for deproteination) were added to 3 mL of the TRF and vortexed for 30 s. The solution was extracted twice with 2 mL hexane. The hexane layers were removed, combined and evaporated in a centrifugal vacuum concentrator (Christ, RVC 2–25 CD plus) and the dried sample re-dissolved in 100 µL acetonitrile and immediately analysed by HPLC.

Both the plasma and TRF samples were analysed using a Shimadzu HPLC (LC-10AD) equipped with a UV–Vis detector (SPD 20A, set at 450 nm). Carotenoids were separated using a ReproSil 80 ODS-2 column (3 µm, 250 × 4.6 mm; Dr. Maisch GmbH, Ammerbuch, Germany) at 40 °C and an eluent in recirculation mode [82% acetonitrile, 15% 1,4-dioxin, and 3% methanol (vol/vol) containing 100 mM ammonium acetate and 10 mM trimethylamine] at a flow rate of 1.5 mL min^−1^ [17]. A β-carotene standard (≥ 97.0% purity, Sigma-Aldrich) was used to construct a standard curve.

### Analysis of triacylglycerols, HDL- and LDL-cholesterol

Plasma TAG, HDL- and LDL-cholesterol were analysed by a clinical laboratory (Laborärzte Sindelfingen, Sindelfingen, Germany).

### Caco-2 cellular uptake assay

The cellular uptake and transepithelial transport (apical to basolateral) of β-carotene from the dietary supplement (β-carotene oil) and a pure synthetic powder (β-carotene powder; Sigma Aldrich, Darmstadt, Germany) was determined using a Caco-2 cell model [18]. In short, Caco-2 cells (HTB-37, ATCC, Wessel, Germany) were cultured in Dulbecco’s Modified Eagle Medium (Sigma-Aldrich) containing 10% foetal bovine serum, 1% sodium pyruvate, 1% nonessential amino acids, and 1% penicillin and streptomycin (all from Carl Roth, Karlsruhe, Germany). Cells were seeded in Transwell membrane inserts (Corning, Kaiserslautern, Germany) at 5 × 10^4^ cells per well and all experiments conducted between passages 12 and 20. The β-carotene oil and β-carotene powder were micellized, using Polysobate 80, according to O'Sullivan et al.[19]. On the day of the experiment, (20–22 days post-confluence) the integrity of the cell monolayer was monitored and all wells with a transepithelial electrical resistance (TEER) less than 250 Ω cm^2^ were discarded. The micellized β-carotene solutions (500 µL) were diluted with modified fasted state simulated intestinal fluid (FaSSIF) transport buffer [20] to a concentration of 100 µM (equivalent to a 15 mg dose taken with 250 mL water), applied to the Caco-2 cells (apical chamber) and incubated for 90 min. To create “sink conditions” in the basolateral compartment, 4% bovine serum albumin was used [21]. To simulate the transport of absorbed compounds in the lymphatic system and away from the intestinal cells, the receiver fluid (1200 μL) in the basolateral chamber was replaced by an equal volume of fresh solution at 15, 30, 45, 60 and 90 min. Finally, the donor fluid in the apical chamber (500 μL), cells (cellular uptake) and pooled basolateral samples (transepithelial transport) were extracted and analysed by HPLC as described for the TRF. In this study cellular uptake and transepithelial transport is defined as the percentage of β-carotene retained by the Caco-2 cells and transported to the basolateral compartment, respectively, as percentage of the total amount of β-carotene applied to the cells.

### β-Carotene solubility and bioaccessibility

To determine the effect of the test meal (breakfast, Table 1) matrix on the bioaccessibility of β-carotene, an in vitro gastrointestinal digestion was performed [22]. The digestive enzymes and bile used in the in vitro digestion; pepsin from porcine gastric mucosa (P7000; ≥ 250 units mg^−1^), pancreatin from porcine pancreas (P7545; 8xUSP specification), porcine bile (B8631) and lipase from porcine pancreas Type II (L3126) were obtained from Sigma-Aldrich. A whole serving of the test meal, with which the β-carotene and mineral supplements were consumed during the human study, was combined and blended into a rough paste. The total weight of the meal was 270 g with a fat content of 6.7% or 18 g (calculated from the nutritional information on the packaging). A simulated digestion of a 15 mg dose β-carotene dose taken together with the whole meal (500 µg β-carotene + 9.0 g meal purée) or with only olive oil (500 µg β-carotene + 0.6 g olive oil + 8.4 g saline) was performed. The olive oil added was equal to the amount of fat in the test meal (6.7% of 9.0 g). The 15 mg β-carotene was either from the supplement (β-carotene oil) or the pure synthetic powder (β-carotene powder).

For each digestion (final volume made up to 25 mL with saline), β-carotene (500 μg) with the meal purée (9.0 g) or olive oil/saline (0.6/8.4 g) was digested with porcine pepsin (4.9 mg mL^−1^; Sigma Aldrich) at a pH of 2.5 for 1 h at 37 °C, shaking at 180 rpm. Porcine pancreatin (1.2 mg mL^−1^), porcine pancreatic lipase (0.6 mg mL^−1^), and porcine bile extract (7.2 mg mL^−1^; all from Sigma Aldrich) were added and the pH adjusted to 6.5. The samples were covered by nitrogen gas and incubated for 2 h, as described above. The digested samples were then centrifuged (13,500×*g*, 60 min, 4 °C), and a subsample of the supernatant was collected and analysed to determine the amount of solubilised β-carotene. The soluble fraction would include bile salt–lipid micelles as well as larger undigested lipid emulsions, which are less physiologically relevant to β-carotene absorption. The remainder of the soluble fraction was filtered (Filtropur S, 0.2 μm, Sarstedt, Nümbrecht, Germany) to separate small bile salt–lipid mixed micelles from the larger lipid emulsions. Both the centrifuged and filtered samples were topped with nitrogen gas and stored at − 80 °C for a maximum of 1 week prior to saponification and HPLC analysis [5]. In this paper, the in vitro solubility is defined as the amount of β-carotene solubilised (supernatant after centrifuging) and bioaccessibility as the amount incorporated into small particles/micelles (supernatant after centrifuging and filtration, 200 nm) after in vitro digestion.

### Statistical analyses

The primary outcomes analysed from the clinical trial were: the area under the plasma concentration–time curve (AUC), using the trapezoid rule, the maximum concentration in plasma (C_max_) and the time to reach the maximum concentration in plasma (T_max_). All data are presented as arithmetic mean ± standard deviation (SD). The results are reported both as measured (Table 2) and baseline-corrected data (Supplemental Table A3). Statistical analyses were calculated using GraphPad Prism 5 (GraphPad Software Inc., San Diego, California, USA). Group means of iron and zinc on blood parameters and differences in β-carotene cellular uptake, transepithelial transport (n = 9), β-carotene solubility and bioaccessibility (n = 4) were compared using one-way ANOVA, with p < 0.05 considered statistically significant.

**Table 2:** Pharmacokinetic variables (mean ± SD) calculated from concentrations of β-carotene in triacylglycerol-rich fraction (TRF) and β-carotene, triacylglycerol (TAG), high density lipoprotein (HDL) and low density lipoprotein (LDL) cholesterol concentrations in plasma (collected every hour for 10 h) of healthy human males after a single oral dose of 15 mg β-carotene together with a placebo, iron sulphate (25 mg iron) or zinc sulphate (30 mg zinc)

	β-Carotene	β-Carotene + iron	β-Carotene + zinc
TRF			
β-Carotene			
AUC (nmol L^−1^ h)	73.4 ± 33.5	76.5 ± 22.3	77.5 ± 37.0
C_max_ (nmol L^−1^)	17.2 ± 10.7	17.1 ± 14.5	18.8 ± 20.6
T_max_ (h)	3.3 ± 4.5	1.8 ± 1.5	2.5 ± 3.2
Plasma			
β-Carotene			
AUC (µmol L^−1^ h)	7.3 ± 3.3	7.9 ± 3.4	7.4 ± 3.3
C_max_ (µmol L^−1^)	0.8 ± 0.4	1.0 ± 0.4	0.8 ± 0.4
T_max_ (h)	5.3 ± 4.4	5.7 ± 3.0	4.3 ± 2.7
TAG			
AUC (mg dL^−1^ h)	1060.0 ± 429.9	1034.0 ± 324.7	973.0 ± 388.0
C_max_ (mg dL^−1^)	153.1 ± 67.2	147.3 ± 43.9	133.3 ± 48.8
T_max_ (h)	7.6 ± 3.3	9.1 ± 1.2	7.4 ± 1.6
HDL-cholesterol			
AUC (mg dL^−1^ h)	474.1 ± 68.4	496.4 ± 75.5	491.6 ± 74.5
C_max_ (mg dL^−1^)	49.4 ± 7.3	52.1 ± 7.8	51.6 ± 7.6
T_max_ (h)	6.6 ± 4.1	6.3 ± 3.2	6.4 ± 4.3
LDL-cholesterol			
AUC (mg dL^−1^ h)	1007.0 ± 248.2	1084.0 ± 251.1	1019.0 ± 230.9
C_max_ (mg dL^−1^)	105.5 ± 25.8	113.7 ± 25.8	107.8 ± 24.8
T_max_ (h)	1.5 ± 2.0	2.7 ± 1.8	2.3 ± 3.9

Differences between groups (n = 6) were analysed for by one-way ANOVA, but no significant differences were observed (p > 0.05)

## Results

### Study population and baseline characteristics

Twelve healthy male participants were enrolled in the study, but only seven volunteers completed the trial. The reasons for terminating the study included personal reasons, a wisdom tooth operation and an ear infection. One of the participants who completed the study was excluded due to high baseline plasma and TRF β-carotene concentrations, indicating non-adherence to the wash-out diet. The anthropometric characteristics and fasting blood chemistry values of the 6 male participants included in the study are displayed in Supplemental Table A1.

### β-Carotene bioavailability

Neither iron, nor zinc supplements taken simultaneously with β-carotene significantly affected β-carotene AUC, C_max_ or T_max_ in plasma or TRF (Table 2). More notable was that, despite the high dose of β-carotene consumed with the test meal (15 mg), there was a negligible postprandial response in plasma and TRF β-carotene concentrations (Fig. 1).

#### Cellular uptake of β-carotene from capsules

We analysed β-carotene in the capsules and found them to contain the amount of β-carotene as displayed on the label (> 7.5 mg all-trans β-carotene/capsule). The β-carotene oil was investigated under a microscope and no crystal-like structures were observed. The capsule also readily dissolved (< 5 min) at a stomach pH of 2.5, releasing the β-carotene oil and making it available for digestion. The Caco-2 cellular uptake and transepithelial transport of micellized β-carotene from the supplement oil (2.41 ± 0.19 & 22.09 ± 7.86%, respectively) and a pure (> 97%) β-carotene powder (3.98 ± 1.21 & 33.63 ± 6.60%, respectively) was then compared (Table 3) and found no significant difference.

**Table 3:** Caco-2 cellular uptake and trans-epithelial transport (mean ± SD) of micellized β-carotene (100 µM) from a supplemental oil or pure powder (> 97%)

	β-Carotene oil	β-Carotene powder
% Cellular uptake	2.41 ± 0.19	3.98 ± 1.21
% Trans-epithelial transport	22.09 ± 7.86	33.63 ± 6.60

Differences between β-carotene oil and powder (n = 9) were analysed for by one-way ANOVA, but no significant differences were observed (p > 0.05)

#### Metabolism of fat from meals consumed

The test meal contained approximately 18 g fat/meal (6.7%, w/w) and a triphasic curve was observed, with peaks at approximately 2 to 3 h after the breakfast, lunch and after afternoon snack, respectively (Fig. 1). The C_max_ of plasma TAG, normalised to the baseline, was 78.7–97.3 mg dL^−1^ (Supplemental Table A3).

#### Possible conversion of β-carotene to retinol

There was no substantial change in the plasma retinol content during the 10 h after the consumption of the supplement and test meal (Fig. 1).

#### β-Carotene bioaccessibility from the test-meal compared to olive oil

The solubility and bioaccessibility of β-carotene from the supplement oil and a > 97% pure β-carotene powder, digested either with the test meal or with olive oil (equal to the amount of fat in the meal), was compared. After digestion with the test matrix, the solubilization (4.46–9.87 μM) and bioaccessibility (1.03–1.07 μM) of β-carotene was only 12–27% and 2.8–2.9%, respectively (Table 4). However, when digested with olive oil, the solubilization (8.74–13.48 μM) and bioaccessibility (4.79–6.42 μM) of β-carotene was 24–36% and 13–17%, respectively. This means that sixfold less β-carotene from the test meal was micellized compared to when it was digested with olive oil.

**Table 4:** Solubilization and bioaccessibility (mean ± SD) of β-carotene (37 µM) from a supplemental oil or pure powder (> 97%) after an in vitro gastro-intestinal digestion with the test meal (breakfast) matrix or olive oil

Digest composition	β-Carotene oil	β-Carotene powder
β-Carotene solubilization (μM)		
Olive oil*	8.74 ± 0.47^b^	13.48 ± 1.96^c^
Test meal**	4.46 ± 0.48^a^ (−47%)	9.87 ± 0.49^b^ (−27%)
β-Carotene bioaccessibility (μM)		
Olive oil	6.42 ± 0.12^b^	4.79 ± 1.86^b^
Test meal	1.03 ± 0.41^a^ (−84%)	1.07 ± 0.49^a^ (−78%)

*Olive oil was added at the same concentration as the fat in the test meal (24 mg mL^−1^ digest)

**See Supplemental Table A2 for test meal (breakfast) composition

^abc^Means of solubilisation/bioaccessibility results across lines and columns with different superscript letters are statistically different at p < 0.05 as analysed by ANOVA (n = 4)

()Differences in β-carotene solubilisation respective to bioaccessibility when digested with the test meal are given as the percentage of that digestion with olive oil

## Discussion

### β-Carotene bioavailability

The lack in β-carotene response could have been caused by multiple factors; (1) the β-carotene from the supplement was not bioavailable, (2) the fat from the test meal was insufficient/ineffective in facilitating β-carotene absorption and/or transport into the lymphatic system, (3) host factors affected the β-carotene absorption or the metabolism and conversion to retinoic acid/retinol during absorption or (4) the test meal matrix decreased the β-carotene bioaccessibility. Below we systematically investigated these possibilities.

#### Cellular uptake of β-carotene from capsules

To determine if the enterocytes could absorb the beta-carotene independent from its bioaccessibility, the supplement was micellized, using polysorbate 80. While the cellular uptake and transepithelial transport of β-carotene from the powder was slightly higher than that from the oil, the difference was not statistically significant and would not explain the lack of response in plasma and TRF β-carotene concentrations.

#### Metabolism of fat from meals consumed

To evaluate the metabolism of fat from the test meal (breakfast) and subsequent meals consumed on the study days, the plasma TAG response was evaluated. The test meal contained enough lipid (18 g fat/meal (6.7%, w/w)) to ensure optimal micellization and absorption of β-carotene [11, 12]. Lipid metabolism is critical to β-carotene metabolism, which includes (1) release from the food matrix, (2) incorporation into physiological mixed micelles, (3) uptake by intestinal epithelial cells, and (4) packaging into chylomicrons that are secreted into the lymphatic system. The triphasic curve confirmed lipid micellization, absorption, and transport into the lymphatic system (Fig. 1) and the high C_max_ of plasma TAG, sufficient lipid bioavailability during the day to ensure β-carotene absorption.

#### β-Carotene metabolism during absorption

Even though the consumption of supplements were an exclusion criterion, it could be possible that the participants still had high vitamin A status, resulting in decreased β-carotene absorption [23]. In humans, between 35 and 90% of absorbed all-trans β-carotene is oxidatively cleaved by the β,β-carotene 15,15’-monooxygenase 1 (BCMO1) into two molecules of all-trans retinal, which subsequently can be oxidised to retinoic acid, or reduced to retinol [13]. There was however no substantial increase in plasma retinol during the 10 h to indicate high conversion (Fig. 1). Dietary factors can affect the activity of BCMO1, resulting in more or less of the absorbed β-carotene being converted [13]. It has been found that higher fat intake increases the post-absorptive conversion of β-carotene. The hepatic vitamin A content of gerbils fed on a 30% fat diet was significantly elevated compared to those fed on a 10% fat diet [24] and vitamin A levels in several tissues (liver, lung, kidney, adipose tissue, and testis) of ferrets fed a diet containing 23% fat was elevated compared with those fed a 6% fat diet [25]. It is possible that the very high fat content of the diet (18 g meal^−1^) could have increased the conversion of the absorbed β-carotene, resulting in decreased post-prandial response.

#### Effect of food matrix factors on β-carotene bioaccessibility

The low bioaccessibility of the β-carotene from the test meal, compare to digestion with olive oil, could be due to food matrix factors.

While fibre is a well-known inhibitor of carotenoid bioavailability [9], the fibre content of < 1% (2.7 g meal^−1^) was lower than the amounts (≈8–10 g) that has been reported to decrease β-carotene bioavailability [26].

The dairy based breakfast test meal (Table 1) also contained approximately 200 mg calcium (calculated from nutritional labelling on packaging), a divalent mineral which has also been found to decreased the micellization and cellular uptake of β-carotene in vitro [4]. While supplementing a meal with up to 1000 mg calcium did not decrease the β-carotene absorption in humans [7], a 500 mg supplement was found to decrease lycopene bioavailability with 83%[6]. While the calcium addition did not decrease the micellization of the lycopene, it did decrease the charge of the physiological mixed micelles.

The fatty acid profile of the dairy-based test meal could have affected the β-carotene micellization efficiency and/or absorption. While the majority of fatty acids in dairy are long chain fatty acids (LCFA; 86%), it also contains substantial amounts of short (SCFA; 5%) and medium chain fatty acids (MCFA; 9%) [27]. The TAG, β-carotene and retinyl-palmitate responses in the TRF of 16 young healthy men were found to be dramatically impaired when β-carotene was consumed with only MCFA compared to with LCFA [28]. This was explained by MCFA not eliciting the chylomicron response required for β-carotene absorption, as MCFA are almost exclusively transported via the portal vein. In the current study, however, the MCFA made up less than 10% of the fat consumed and the substantial plasma TAG response observed (Supplemental Tables A1 and A3) suggests that enough chylomicrons were produced to facilitate, if not all, a substantial amount of β-carotene transport into the lymphatic system.

When digested with the test meal, compared to olive oil, β-Carotene bioaccessibility was decreased more (78–84%), than β-carotene solubilisation (27–47%) (Table 4), suggesting that larger lipid vesicles formed, which were removed after filtration (200 nm). In the study by Borel et al. [28] decreased TRF β-carotene concentration after consumption of a MCFA vs. a LCFA rich meal was attributed to less β-carotene being absorbed into the enterocytes, because less was packaged into MCFA micelles compared to LCFA micelles. Salvia-Trujillo et al. [29] evaluated the effect of the fatty acid chain length on the in vitro bioaccessibility of β-carotene. They also observed a sixfold decrease in in vitro bioaccessibility of β-carotene when the lipid profile was changed from 100% LCFA to 100% MCFA. This was attributed to the easier incorporation of the long chain non-polar β-carotene into micelles formed by LCFA. They also proposed that β-carotene digested with LCFA is better solubilized within the mixed micelles, because during digestion with MCFA, β-carotene is trapped within larger vesicles that are less likely to be absorbed by enterocytes [30].

A large proportion (60%) of the total protein (14 g) in the meal was from the dairy products (yoghurt and cream cheese), which could have decreased the β-carotene solubilisation and subsequent bioaccessibility. Proteins play an important and varied effect in β-carotene bioavailability. While low protein status has been found to decrease intestinal cleavage of β-carotene [31], the low bioavailability of β-carotene from plant sources has, in part, been attributed to the it being bound to intrinsic plant proteins[32], which is not the case with Supplements. It is however possible that the proteins from the food matrix could interact with the supplemental β-Carotene during digestion [33]. In a study evaluating the food matrix effect on β-Carotene bioaccessibility, the β-Carotene bioaccessibility from fat-free yogurt was found to be fivefold lower than that from a fat-free tapioca pudding [34]. This was attributed to (1) coagulation of the yogurt proteins during the digestion, (2) the higher protein content of the yogurt, which decreased the solubilisation of β-carotene into the lipid phase and (3) the high casein content, which has been found to bind bile salts, decreasing the extent of micelle formation [9]. In rats it has also been found that soluble casein proteins can inhibit the incorporation of β-carotene into micelles, possibly due reducing enzyme access to the lipid droplets[35].

## Concluding remarks

Despite the limitations of the human study (small number of participants and lack of post-prandial β-carotene response), the work still provides valuable insights into the possible role of the food matrix factors in β-carotene bioaccessibility and post-prandial conversion. It also provides important information for the successful planning of future studies. The dairy-based food matrix seems to have played a significant role in the low bioavailability of the β-carotene from the test-meal. First, the very high fat content of a meal could result in a high post-absorptive conversion of β-carotene to retinol. Secondly, while no constituent of the meal alone (high fat content, MCFA, fibre, calcium or casein) fully explains the lack of response in TRF β-carotene, the combined effects and/or interactions between some of the constituents could. The interactions of β-carotene and modulators of its bioavailability within dairy-based food matrices require more research.

## Supplementary Information

Below is the link to the electronic supplementary material.

Supplementary file1 (PDF 150 KB)

## Abbreviations

TRF: Triacylglycerol rich fraction
TAG: Triacylglycerol
FaSSIF: Fasted state simulated intestinal fluid
AUC: Area under the plasma concentration–time curve
C_max_: Maximum concentration in plasma
T_max_: Time to reach the maximum concentration in plasma
LCFA: Long chain fatty acids
SCFA: Short chain fatty acids
MCFA: Medium chain fatty acids
SFA: Saturated fatty acids m
MUFA: Monounsaturated fatty acids
PUFA: Polyunsaturated fatty acids
BCMO1: β,β-Carotene 15,15’-monooxygenase 1
