# Supplementary material for: Low β-carotene bioaccessibility and bioavailability from high fat, dairy-based meal
Source: Eur J Nutr. 2024 May 16;63(6):2261–70. doi: 10.1007/s00394-024-03423-w (PMC11377643; doi:10.1007/s00394-024-03423-w)
Supplement: Supplementary file 1 — Supplementary file1 (PDF 150 KB) [file 394_2024_3423_MOESM1_ESM.pdf]

Supporting Information

Table S1: Anthropometric characteristics and fasting blood chemistry values of the 6 male participants whose results were included in the final analysis

Parameter	*Reference range*	*Participants (n=6)*
Anthropometric measurements		
Age (years)	18-45	25 ± 4
Body weight (kg)	-	74.3 ± 12.7
Body height (m)	-	1.80 ± 0.1
BMI (kg/m²)	19 - 25	22.3 ± 1.6
Systolic blood pressure (mmHg)	< 130	116.5 ± 10.4
Diastolic blood pressure (mmHg)	< 90	79.8 ± 6.7
Lipids		
Total cholesterol (mg/dL)	< 200	177.6 ± 27.3
LDL cholesterol (mg/dL)	< 155	113.9 ± 20.7
HDL cholesterol (mg/dL)	35-100	54.0 ± 7.6
LDL/HDL cholesterol ratio	0.5 - 3.5	2.2 ± 0.6
Triglycerols (mg/dL)	< 200	94.3 ± 54.5
Kidney and liver function markers		
γ-GT (U/L)	< 60	23.2 ± 11.6
AST (U/L)	< 50	32.8 ± 10.2
ALT (U/L)	< 50	25.0 ± 9.3
Alkaline phosphatase (U/L)	40 - 130	76.2 ± 13.1
Creatinine i.S. (mg/dL)	0.70 – 1.20	0.9 ± 0.1
Bilirubin (mg/dL)	< 1.10	0.8 ± 0.5
Uric acid (mg/dL)	< 7	5.1 ± 0.9

Table S2: List of allowed foods during three-day strict diet of the washout week

**Vegetables:**	**Fruits:**
Potatoes	Lychee
Mushrooms	Coconut
Garlic	**Drinks:**
White onions	Coffee
Celery root	Water
Cauliflower	White wine
White asparagus	Beer
**Others:**	Low-fat milk
Sunflower oil	**Meat / protein rich foods:**
Olive oil	Pork meat
Nuts (except pistachios)	Beef
Low-fat milk and dairy products	Chicken
Soy milk	Sausage
Coconut milk	Beef extract
Almond milk	Soy products
Low-fat natural yoghurt	White beans
Low-fat cream cheese	**Snacks:**
Low-fat white cheese (feta cheese, brie, camembert)	Chocolate
Mayonnaise	Nuts
Pickled white onions	Salted potato Chips
Peanut butter	**Grain products:**
Chocolate spread	White noodles (pasta)
Honey	Wheat flour
Syrup	White rice
Salt	Waffles
White pepper	Bread (no cornmeal-bread)
Black pepper	

Table S3: Pharmacokinetic variables (mean ± SD) calculated from baseline normalised concentrations of β-carotene in triacylglycerol-rich fraction (TRF) and β-carotene, triacylglycerol (TAG), high-density lipoprotein (HDL) and low-density lipoprotein (LDL) cholesterol concentrations in plasma of healthy human males after a single oral dose of 15 mg β-carotene together with a placebo, iron sulphate (25 mg iron) or zinc sulphate (30 mg zinc).

	β-Carotene	β-Carotene + iron	β-Carotene + zinc
	Triacylglycerol-rich fraction (TRF)		
β-Carotene			
AUC (nmol/L · h)	-0.12 ± 0.52	0.21 ± 0.71	-0.09 ± 0.23
C_{max} (nmol/L)	0.10 ± 0.14	0.20 ± 0.21	0.06 ± 0.04
T_{max} (h)	5.33 ± 4.41	5.67 ± 3.01	4.33 ± 2.66
	Plasma		
β-Carotene			
AUC (µmol/L · h)	-29 ± 32.5	-24 ± 16.2	-28 ± 11.7
C_{max} (µmol/L)	-4.1 ± 7.6	-0.8 ± 5.2	-2.9 ± 5.6
T_{max} (h)	5.17 ± 2.86	4.83 ± 2.32	5.83 ± 3.37
TAG			
AUC (mg/dL · h)	457.8 ± 250.5	402.9 ± 218.4	402.7 ± 253.8
C_{max} (mg/dL)	97.3 ± 48.3	88.0 ± 29.2	78.7 ± 34.3
T_{max} (h)	8.83 ± 1.84	9.33 ± 1.21	7.67 ± 1.63
HDL-cholesterol			
AUC (mg/dL · h)	4.1 ± 12.2	-1.5 ± 8.9	-8.4 ± 16.3
C_{max} (mg/dL)	2.43 ± 0.98	2.14 ± 1.46	1.71 ± 1.38
T_{max} (h)	6.57 ± 4.08	6.00 ± 3.61	6.29 ± 4.19
LDL-cholesterol			
AUC (mg/dL · h)	5.08 ± 8.05	3.20 ± 2.32	6.25 ± 10.33
C_{max} (mg/dL)	2.29 ± 3.4	2.43 ± 1.62	3.00 ± 4.20
T_{max} (h)	3.57 ± 3.26	4.86 ± 2.34	4.00 ± 3.83

Differences between groups (n=6) were analysed for by one way ANOVA, but no significant differences were observed

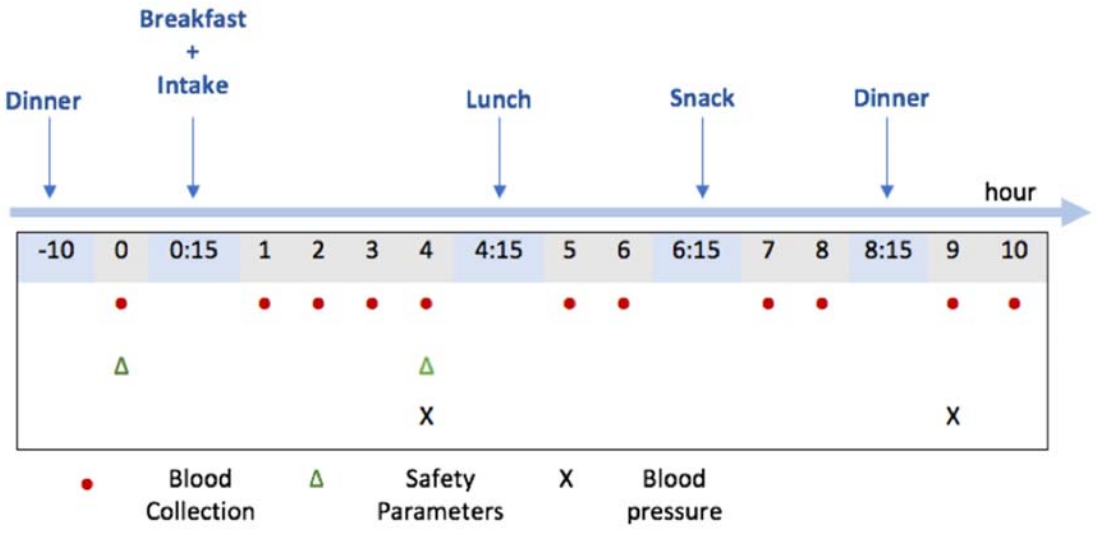

Figure S1: Scheme of the time points of meal intake, blood draws, and samples taken during each intervention day
